# Measuring cystic fibrosis drug responses in organoids derived from 2D differentiated nasal epithelia

**DOI:** 10.26508/lsa.202101320

**Published:** 2022-08-03

**Authors:** Gimano D Amatngalim, Lisa W Rodenburg, Bente L Aalbers, Henriette HM Raeven, Ellen M Aarts, Dounia Sarhane, Sacha Spelier, Juliet W Lefferts, Iris AL Silva, Wilco Nijenhuis, Sacha Vrendenbarg, Evelien Kruisselbrink, Jesse E Brunsveld, Cornelis M van Drunen, Sabine Michel, Karin M de Winter-de Groot, Harry G Heijerman, Lukas C Kapitein, Magarida D Amaral, Cornelis K van der Ent, Jeffrey M Beekman

**Affiliations:** 1 Department of Pediatric Pulmonology, Wilhelmina Children’s Hospital, University Medical Center Utrecht, Utrecht University, Member of ERN-LUNG, Utrecht, The Netherlands; 2 Regenerative Medicine Center Utrecht, University Medical Center Utrecht, Utrecht University, Utrecht, The Netherlands; 3 Department of Pulmonology, University Medical Center Utrecht, Utrecht, The Netherlands; 4 BioISI-Biosystems and Integrative Sciences Institute, Faculty of Sciences, University of Lisboa, Lisboa, Portugal; 5 Department of Biology, Cell Biology, Neurobiology and Biophysics, Faculty of Science, Utrecht University, Utrecht, The Netherlands; 6 Centre for Living Technologies, Eindhoven-Wageningen-Utrecht Alliance, Utrecht, The Netherlands; 7 Department of Otorhinolaryngology, Amsterdam University Medical Centers, University of Amsterdam, Amsterdam, The Netherlands

## Abstract

Cystic fibrosis is caused by genetic defects that impair the CFTR channel in airway epithelial cells. These defects may be overcome by specific CFTR modulating drugs, for which the efficacy can be predicted in a personalized manner using 3D nasal-brushing–derived airway organoids in a forskolin-induced swelling assay. Despite of this, previously described CFTR function assays in 3D airway organoids were not fully optimal, because of inefficient organoid differentiation and limited scalability. In this report, we therefore describe an alternative method of culturing nasal-brushing–derived airway organoids, which are created from an equally differentiated airway epithelial monolayer of a 2D air–liquid interface culture. In addition, we have defined organoid culture conditions, with the growth factor/cytokine combination neuregulin-1*β* and interleukin-1*β*, which enabled consistent detection of CFTR modulator responses in nasal-airway organoid cultures from subjects with cystic fibrosis.

## Abstract (precis)

This work describes an optimized assay to determine CFTR drug responses in people with cystic fibrosis, using nasal-airway organoids that are generated from 2D differentiated epithelial monolayers.

## Introduction

Cystic fibrosis (CF) is a monogenic epithelial disease caused by mutations in the *CFTR* gene (Riordan et al, 1989). This defect impairs CFTR-dependent anion conductance in airway epithelia (Knowles et al, 1983), which leads to a severe respiratory disease (Stoltz et al, 2015). CFTR modulators are target-specific drugs that may restore CFTR function in individuals with CF (Boyle & De Boeck, 2013). However, the efficiency of modulators largely depends on the CFTR genotype of an individual with CF. More than 2,000 distinct *CFTR* mutations have been reported (http://www.genet.sickkids.on.ca//) with variable effects on CFTR expression or function. In addition to common mutations, such as the F508del allele, ∼1,000 rare mutations have been identified that each affects less than five individuals worldwide. This low prevalence makes it unfeasible to determine CFTR modulator drug efficacy in large cohort clinical studies.

As an alternative of determining drug efficacy directly in individuals with CF, the effects of CFTR modulators can be predicted using patient-derived epithelial cultures in functional CFTR assays (Clancy et al, 2019). This is traditionally done with 2D air–liquid interface (ALI)–differentiated airway epithelia by assessment of CFTR-dependent chloride (Cl^−^) conductance via electrophysiology (Awatade et al, 2015; Gentzsch et al, 2017; Pranke et al, 2017). However, a major disadvantage of the ALI culture model system is the limited scalability. In contrast, CFTR-expressing epithelial organoids from various tissues, that is, the airway, intestine, and kidney, are emerging as a novel model system in which drug efficacy can be tested more efficiently in a mid- to high-throughput fashion (Dekkers et al, 2013; Sachs et al, 2019; Schutgens et al, 2019). Previously, we and others have shown that intestinal organoids from subjects with CF can be used to predict drug responses in a forskolin-induced swelling (FIS) assay, reflecting CFTR-dependent fluid secretion (Dekkers et al, 2016; Berkers et al, 2019; Ramalho et al, 2020; van Mourik et al, 2019). Nevertheless, based on the origin of CF respiratory disease, it remains postulated that airway epithelial models are more predictive for determining CFTR modulator responses.

Indeed, we previously reported CFTR modulator response measurements in long-term expanded distal airway organoids using FIS (Sachs et al, 2019). However, we observed large variations in FIS measurements as swelling was limited to well-differentiated spherical structures. Others have successfully used 3D nasal-airway organoids (NAOs) derived from minimal-invasive nasal brushings in functional CFTR assays (Guimbellot et al, 2017; Brewington et al, 2018; Liu et al, 2020), which are a more suitable option for personalized drug testing compared with cultures derived from invasive (tracheo)bronchial and intestinal tissues. However, previously described functional CFTR assays using NAOs were low in throughput (Guimbellot et al, 2017; Brewington et al, 2018; Liu et al, 2020; Sette et al, 2021). Therefore, there is a remaining need for a further optimized and scalable FIS assay using airway organoids, especially derived from nasal brushings, which enables CFTR modulator response measurements in subjects with CF. In this report, we describe an alternative organoid culture method, in which NAOs are derived from 2D differentiated human nasal epithelial cell (HNEC) monolayers. We furthermore describe optimized airway organoid culture condition to improve CFTR modulator response measurements, by including the growth factor/cytokine combination neuregulin-1*β* and IL-1*β*. Validation studies using this culture condition showed consistent detection of genotype-specific responses to CFTR modulators in FIS assays, including repairing effects of the FDA-approved CFTR triple modulator therapy VX-661/VX-445/VX-770 (Keating et al, 2018).

## Results and Discussion

**Figure S1.**
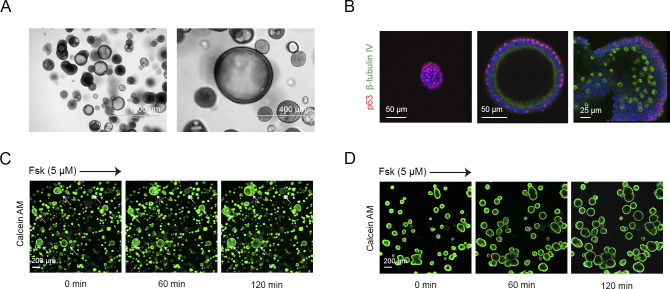
Heterogeneity in passaged tissue-derived airway organoid cultures. **(A)** Brightfield images of airway organoids, passaged after mechanical disruption according to the method described by Sachs et al (2019), showing differences in organoid morphology, that is, structures with lumen and solid colonies. **(B)** Immunofluorescence confocal imaging of airway organoids from the same culture, stained for the basal-cell marker p63 (red) and ciliated-cell marker *β*-tubulin IV (green). Image’s show (left) a solid colony consisting solely of p63^+^ basal cells, (middle) a pseudostratified structure with lumen but without ciliated cells, and (right) a large structure displaying ciliated cells. **(C)** Representative images (t = 0, 60, and 120 min after 5 μM forskolin stimulation) of a forskolin-induced swelling assay using serial passaged HC airway organoids stained with calcein green AM. Arrows indicate swelling of large-sized structures. **(D)** Representative images (t = 0, 60, and 120 min after 5 μM forskolin stimulation) of a forskolin-induced swelling assay using HC airway organoids derived from an air–liquid interface culture stained with calcein green AM.

**Figure 1.**
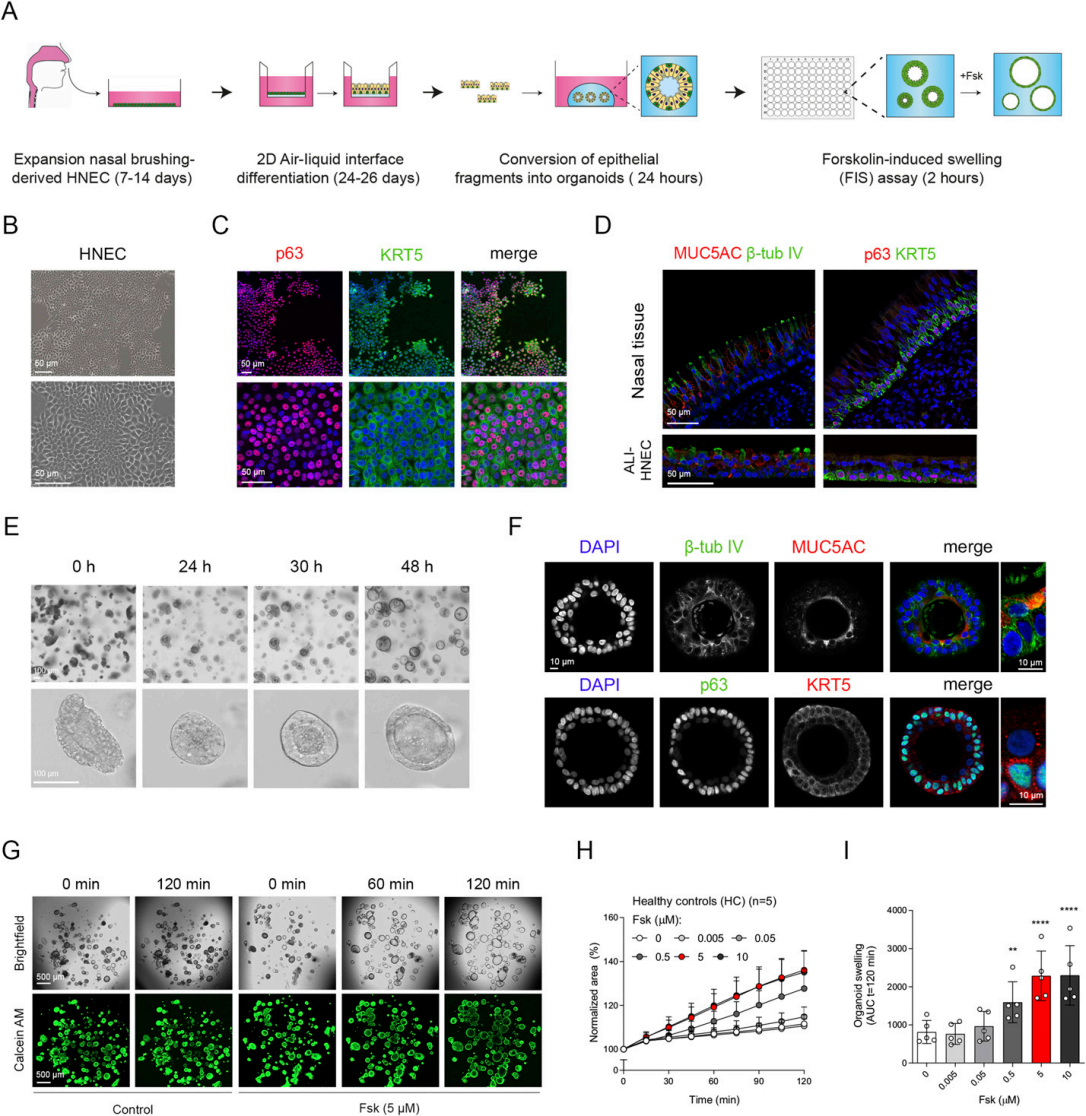
Conversion of differentiated air–liquid interface (ALI) human nasal epithelial cell (HNEC) cultures into nasal-airway organoids (NAOs) and use in forskolin-induced swelling (FIS) assays. **(A)** Graphic illustration showing workflow of culturing NAOs from 2D differentiated HNECs and use in FIS assays. **(B)** Brightfield images of HNEC. **(C)** IF staining of HNEC with basal-cell markers p63 (red) and cytokeratin 5 (KRT5, green). **(D)** Sections of nasal tissue (top) and 18 d differentiated ALI-cultured HNEC (bottom), demonstrating IF staining of MUC5AC (goblet cells, red), *β*-tubulin IV (ciliated cells, green), p63, and KRT5 (basal-cell markers, green and red, respectively). **(E)** Time course showing self-organization of differentiated ALI-HNEC–derived epithelial fragments into organoids. **(F)** Confocal images showing in the top panels staining with DAPI (blue), *β*-tubulin IV (green), and MUC5AC (red). The bottom panels show staining with DAPI (blue), p63 (green), and KRT5 (red). **(G)** Representative brightfield images (top) and images of calcein green AM esters–stained (bottom) organoids from a HC subject, which were unstimulated (control) or treated with forskolin (Fsk, 5 μM). Images were taken at t = 0, 60, and 120 min after stimulation. **(H, I)** NAOs from HC subjects (n = 5 independent donors) were stimulated with different concentrations of Fsk (0–10 μM), followed by quantification of FIS. **(C, D, F)** Data information: DAPI (blue) was used as nuclear staining (C, D, F). **(H, I)** Quantification of FIS is depicted as the percentage change in surface area relative to t = 0 (normalized area) measured at 15-min time intervals for 2 h (means ± SD) (H), and area-under-the-curve (AUC) plots (t = 120 min, means ± SD, datapoints represent individual donors) (I). **(I)** Analysis of differences was determined with a one-way ANOVA and Bonferroni post hoc test (I). **P < 0.01, ****P < 0.0001.

**Figure S2.**
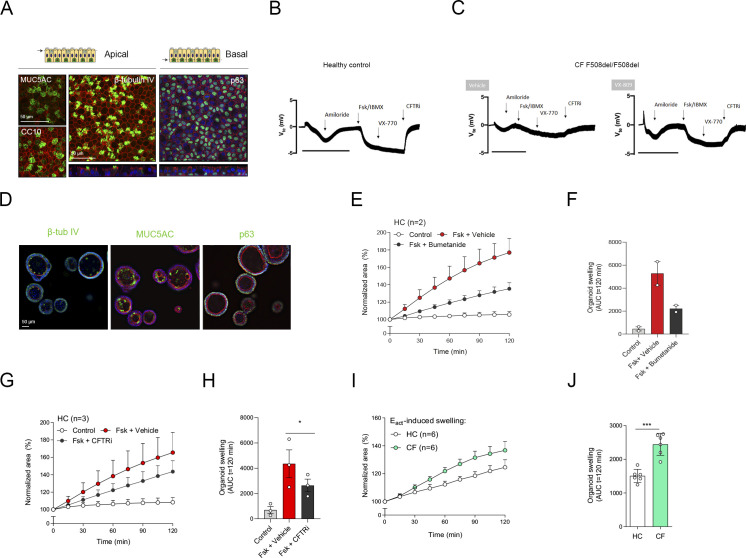
Characterization of air–liquid interface (ALI)-human nasal epithelial cell (HNEC) and nasal-airway organoid (NAO) swelling assay. **(A)** Whole-mount IF confocal images of ALI-HNEC, showing maximal projections of the apical side of the secretory cell markers MUC5AC and club cell protein 10 (CC10), and ciliated cell marker *β*-tubulin IV (Z-stack at the bottom). The projection of the basal side shows the basal-cell marker p63 (Z-stack at the bottom). All markers are shown in green. **(B, C)** Open circuit Ussing chamber tracings showing transepithelial voltage measurements (V_te_) from healthy control (B) and cystic fibrosis (CF) F508del/F508del (C) ALI-HNEC cultures. CF cultures were pre-treated with VX-809 or vehicle. During measurements, cells were treated sequentially with amiloride, Fsk/IBMX, VX-770, and CFTRi. The bar corresponds to a 10-min frame. **(D)** IF staining of NAOs for *β*-tubulin IV, MUC5AC, and p63 (all in green). **(E, F)** HC NAOs (n = 2 independent donors) were pre-treated with bumetanide or vehicle for 4 h, followed by stimulation with forskolin (Fsk) and assessment of forskolin-induced swelling (FIS). **(G, H)** HC NAOs (n = 3 independent donors) were pre-treated with CFTR inhibitors or vehicle, followed by assessment of FIS. **(I, J)** Comparison between HC and CF organoid swelling (both n = 6 independent donors) after stimulation with the calcium-activated chloride channel agonist E_act_. **(A, B)** Data information: DAPI (blue) was used as nuclear staining; phalloidin (red) was used as actin cytoskeleton staining (A, D). **(E, F, G, H, I, J)** FIS assay results are depicted as the percentage change in surface area relative to t = 0 (normalized area) measured at 15-min time intervals for 2 h (means ± SD) (E, G, I) and as area-under-the-curve (AUC) plots (t = 120 min, means ± SD) (F, H, J). **(H, J)** Analysis of differences was determined with a paired (H) or unpaired (J) *t* test. *P < 0.05, ***P < 0.001.

We previously reported CFTR function measurements in distal airway organoids in FIS assays (Sachs et al, 2019). Upon passaging of mechanically disrupted organoids, we observed however large variation in organoid morphology, corresponding with differences in differentiation of individual organoid structures within the same culture (Fig S1A and B). CFTR function is mediated by differentiated cells. Therefore, this unsynchronized organoid differentiation reduces the accuracy of quantifying FIS, which is based on measuring the total surface area increase of all organoids within a single culture well (Fig S1C and Video 1). To generate evenly differentiated airway organoids that display FIS (Fig S1D and Video 2), we set up a method in which organoids are established from a 2D differentiated HNEC monolayer (Fig 1A). In the culture procedure, HNEC derived from nasal brushings were first isolated and expanded in regular 2D cell cultures (Fig 1B). HNEC stained positive for p63 and cytokeratin 5 (KRT5), confirming a basal–stem cell phenotype (Fig 1C). After expansion, HNEC were cryopreserved as a master (passage 2) and working (passage 3) cell bank to enable repeated usage of donor materials (detailed description in the Materials and Methods section). HNECs expanded from a working cell bank were subsequently differentiated in conventional 2D ALI transwell cultures, to recapitulate the mucociliary airway epithelium. Similar to the native nasal epithelial tissue (Fig 1D), ALI-HNEC cultures displayed a pseudostratified morphology and consisted of all major airway epithelial subsets, that is, MUC5AC/CC10^+^ secretory cell, *β*-tubulin IV^+^ ciliated cells, and p63/KRT5^+^ basal cells (Figs 1D and S2A). Further studies are required to determine the presence of rare cell types, such as ionocytes (Montoro et al, 2018; Plasschaert et al, 2018). CFTR function and effects of modulators were confirmed in ALI-HNEC from a healthy control (HC) and individual with CF and a F508del/F508del genotype (Fig S2B and C). During isolation of airway organoids from resected airway tissues, based on the method described by Sachs et al (2019), we observed that large epithelial fragments, obtained after collagenase treatment, self-organized into differentiated organoids within a few days after gel embedding. Based on this observation, we proposed that epithelial fragments from 2D-differentiated ALI cultures could be converted into 3D organoids as well. Indeed, embedding of differentiated ALI-derived epithelial fragments in a 3D extracellular matrix led to formation of organoids within 24 h, with visible lumens formation within 48 h (Fig 1E). From a single 12-mm transwell insert, we are able to generate a yield of organoids that is sufficient for 48 independent wells (∼25–50 organoids/well) of a 96-well plate. In terms of scalability and cost efficiency, this demonstrates a major advantage compared with the conventional use of 2D ALI cultures. ALI culture–derived NAOs directly displayed a differentiated phenotype. This was confirmed by visual observation of beating cilia and accumulation of mucus (Video 3), as well as by immunofluorescence imaging, demonstrating *β*-tubulin IV^+^ cilia and MUC5AC^+^ secretory cells inside of the organoids and p63^+^/KRT5^+^ basal cells at the basal side (Figs 1F and S2D).

Video 1 Live imaging of forskolin-induced swelling measured in serial passaged nasal-airway organoids. Video showing forskolin-induced swelling measured in serial passaged healthy control nasal-airway organoids, showing only swelling of a few large organoids within the same culture. Download video

Video 2 Live imaging of forskolin-induced swelling measured in air–liquid interface culture–derived nasal-airway organoids. Video showing forskolin-induced swelling measured in HC nasal-airway organoids, showing equal swelling of all organoids within the same culture. Download video

Video 3 Live imaging of an air–liquid interface culture–derived nasal-airway organoid. Video recording of a cystic fibrosis nasal-airway organoid showing beating cilia at the luminal side of the structure and accumulated mucus. Download video

**Figure 2.**
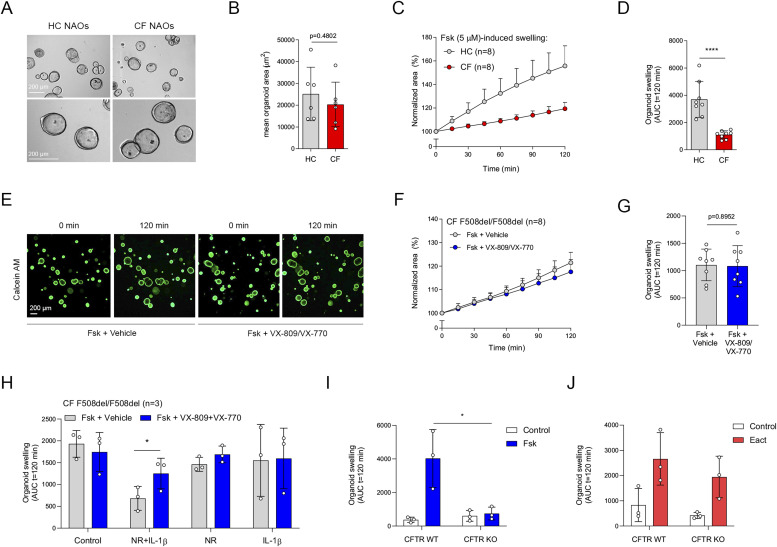
Comparison between HC and cystic fibrosis (CF) nasal-airway organoids (NAOs) and validation of NR/IL-1*β* organoid culture conditions. **(A)** Representative brightfield images of HC and CF NAOs. **(B)** Quantification of the mean organoid area (μm^2^, means ± SD) of HC and CF NAOs (both n = 6 independent donors). **(C, D)** Comparison of forskolin-induced swelling (FIS) between HC and CF NAOs (both n = 8 independent donors) after forskolin (Fsk, 5 μM) stimulation. **(E)** Representative images of calcein green AM esters–stained organoids from a CF F508del/F508del subject, treated with vehicle or VX-809/VX-770. Images were taken at t = 0 and 120 min after stimulation with Fsk. **(F, G)** CF F508del/F508del NAOs (n = 8 independent donors) were pre-treated with VX-809 or vehicle control for 48 h. Subsequently, FIS was determined after acute stimulation with Fsk, together with VX-770 or vehicle control. **(H)** CF F508del/F508del NAOs (n = 3 independent donors) were cultured at control conditions or with NR, IL-1*β* or combination (NR+IL-1*β*). Afterward, FIS was determined in response to VX-809/VX-770. **(I, J)** FIS and (J) E_act_.-induced swelling measured in CFTR WT or KO NAOs (n = 3 independent donors). **(C, D, F, G, H, I, J)** Data information: Results of organoid swelling are depicted as the percentage change in surface area relative to t = 0 (normalized area) measured at 15-min time intervals for 2 h (means ± SD) (C, F) and area-under-the-curve (AUC) plots (t = 120 min, means ± SD, datapoints represent individual donors) (D, G, H, I, J). **(B, D, G, H, I)** Analysis of differences was determined with an unpaired *t* test (B, D, G, I) and one-way ANOVA and Bonferroni post hoc test (H). *P < 0.05, ****P < 0.0001.

Next, we determined whether ALI-derived NAOs could be used to measure CFTR function in FIS assays in a 96-well plate format. Forskolin (Fsk) stimulation of organoids from HC subjects increased swelling in time, which was dose-dependent and reached a plateau at a concentration of 5 μM (Fig 1G–I). Organoid swelling was partly attenuated with the Na(+)-K(+)-Cl(−) cotransporter (NKCC1) inhibitor bumetanide, demonstrating Cl^−^ dependence of forskolin-induced organoid fluid secretion (Fig S2E and F). Lack of complete inhibition may be explained by reabsorption and recycling of luminal-secreted Cl^−^ or an ion channel–independent mechanism underlying fluid secretion caused by mechanical forces (Bajko et al, 2020; Buddington et al, 2021). Moreover, chemical CFTR inhibitors significantly reduced FIS in HC NAOs (Fig S2G and H), indicating CFTR dependence. Upon comparison of cultures from HC subjects and subject with CF, we observed that both HC and CF NAOs displayed cystic lumens, which were not significant different in size (Fig 2A and B). This corresponded with observations made in distal airway organoids (Sachs et al, 2019) and suggests intrinsic CFTR-independent fluid secretion mediated by alternative Cl^−^ channels. In line with this observation, stimulation with the Ca^2+^-activated Cl^−^ channel (CaCC) activator E_act_-induced organoid swelling in CF NAOs, which was significantly higher compared with HC cultures (Fig S2I and J). This further suggests dominant CFTR-independent fluid secretion in CF NAOs, also observed by others in Ussing chamber measurements in ALI cultures (Clarke & Boucher, 1992).

**Figure S3.**
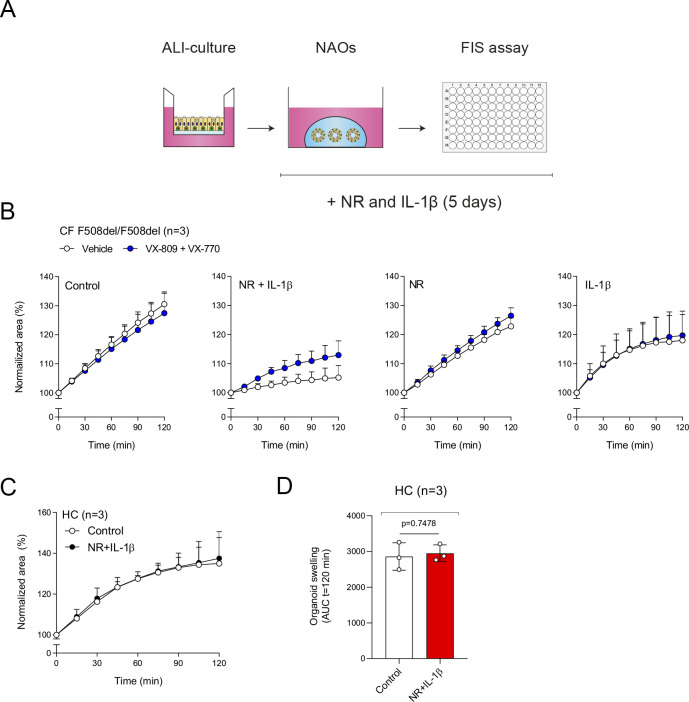
Optimization of CFTR modulator responses in nasal-airway organoids (NAOs) with NR/IL-1*β*. **(A)** Illustration showing culturing of NAOs with neuregulin 1*β* (NR) and interleukin-1*β* (IL-1*β*). **(B)** NAOs from F508del homozygous subjects with cystic fibrosis (n = 3 independent donors) were cultured without additional stimuli (control), with neuregulin (NR), interleukin-1*β* (IL-1*β*), or combination of both (NR+IL-1*β*) for 5 d. At day 3, cultures were pre-treated with VX-809 or vehicle control for 48 h and afterward stimulated with forskolin (Fsk, 5 μM), in combination with VX-770 or vehicle control. **(C, D)** Assessment of forskolin-induced swelling in HC NAOs (n = 3 independent donors) cultured under control conditions or NR+IL-1*β*. **(B, C, D)** Forskolin-induced swelling assay results are depicted as the percentage change in surface area relative to t = 0 (normalized area) measured at 15-min time intervals for 2 h (means ± SD) (B, C) and as area-under-the-curve (AUC) plots (t = 120 min, means ± SD) (D). **(D)** Analysis of differences was determined with a paired *t* test (D).

**Figure S4.**
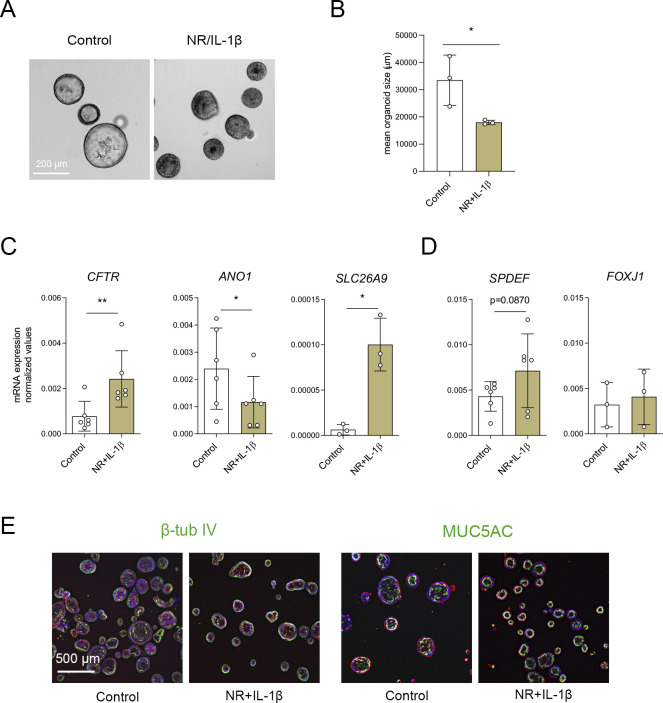
Characterization of nasal-airway organoids (NAOs) cultured with NR/IL-1*β*. **(A)** Brightfield images of cystic fibrosis (CF) F508del/F508del NAOs cultured in control conditions or with NR+IL-1*β* for 5 d. **(B)** Quantification of the mean organoid area (μm^2^, means ± SD) of control and NR+IL-1*β* cultured CF F508del/F508del NAOs (n = 3 independent donors). **(C, D)** mRNA expression analysis of CF F508del/F508del NAOs (n = 3–6 independent) that were left unstimulated (control) or cultured with NR+IL-1*β* for 5 d. **(C, D)** mRNA expression was determined of (C) *CFTR*, *ANO1*, *SLC26A9* (all chloride channels), (D) *SPDEF* (secretory cells), and *FOXJ1* (ciliated cells). Results represent target mRNA expression normalized for the geometric mean expression of the housekeeping genes *ATP5B* and *RPL13A* (means ± SD). **(E)** Immunofluorescence staining of CF organoids (control and cultured with NR+IL-1*β* for 5 d) was conducted of *β*-tubulin IV (ciliated cell) and MUC5AC (goblet cell) (green). DAPI (blue) was used for nuclear staining. Phalloidin (red) was used for actin cytoskeleton staining. **(B, C, D)** Analysis of differences was determined with a paired *t* test (B, C, D). *P < 0.05, **P < 0.01.

**Figure S5.**
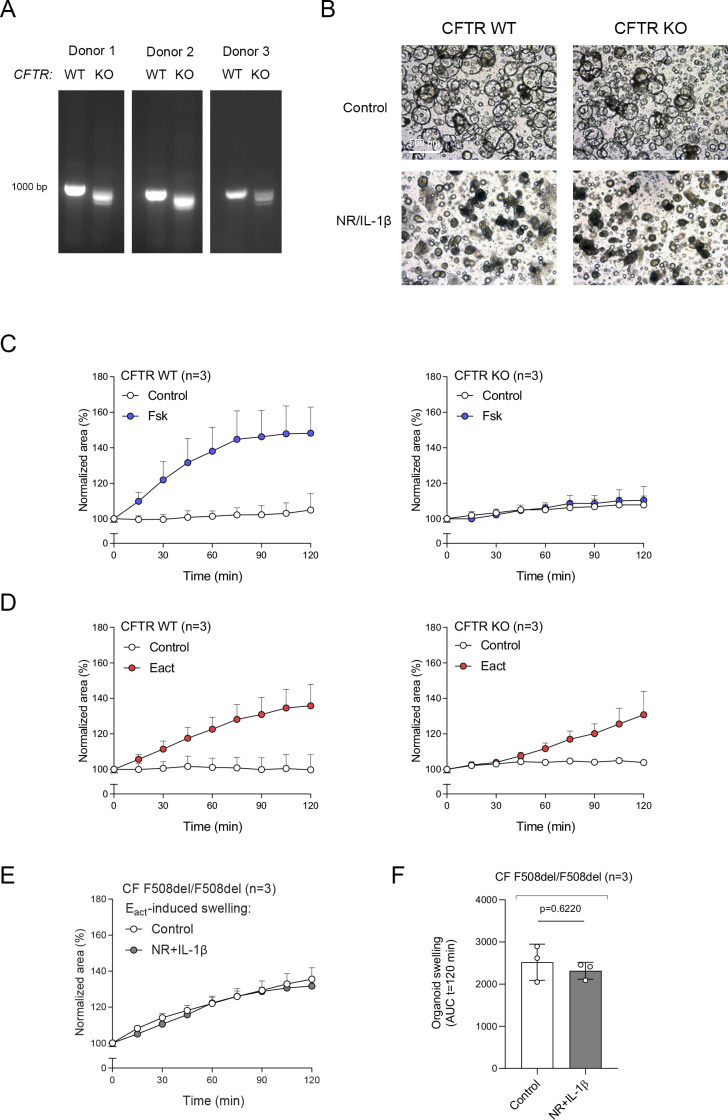
Validation of organoid swelling in CFTR KO cells generated with CRISPR-based gene editing. **(A)** Confirmation of CFTR gene KO (n = 3 independent donors) by agarose gel electrophoresis. **(B)** Brightfield images of CFTR WT and KO nasal-airway organoids (NAOs) cultured in control conditions or with NR+IL-1*β* for 5 d. **(C, D)** Forskolin-induced swelling and (D) E_act_.-induced swelling measured in CFTR WT or KO NAOs (n = 3 independent donors). **(E, F)** E_act_.-induced swelling in cystic fibrosis F508del/F508del NAOs (n = 3 independent donors). **(C, D, E, F)** Organoid swelling results are depicted as the percentage change in surface area relative to t = 0 (normalized area) measured at 15-min time intervals for 2 h (means ± SD) (C, D, E) and as area-under-the-curve (AUC) plots (t = 120 min, means ± SD) (F). Analysis of differences was determined with a paired *t* test.

FIS measurements in NAOs from HC subjects were significantly higher compared with subjects with CF (Fig 2C and D). Although CF and HC NAOs could be distinguished phenotypically based on FIS, we were unable to observe repairing effects of the CFTR corrector and potentiator combination VX-809/VX-770 in CF F508del/F508del NAOs (Fig 2E–G), likely because of dominant CFTR-independent fluid secretion and low CFTR expression. Therefore, we aimed to optimize CFTR modulator response measurements by modifying organoid culture conditions. Recent studies suggested that secretory cells are the primary-airway epithelial-cell type, mediating CFTR function (Carraro et al, 2021; Okuda et al, 2021). Therefore, we examining a panel of growth factors and cytokines that could modulate secretory cell functions, added after plating of the epithelial fragments and during organoid culturing (Fig S3A). The examined factors included, neuregulin-1*β* (NR), which has been reported to enhance epithelial polarization and differentiation of secretory cells in ALI cultures (Vermeer et al, 2006; Kettle et al, 2010). Moreover, the effect of the pro-inflammatory cytokine IL-1*β* was examined, which has been shown to enhance goblet cell differentiation, *CFTR* mRNA expression, Cl^−^ conductance, and CFTR modulator responses in ALI cultures (Brouillard et al, 2001; Gray et al, 2004; Abdullah et al, 2018; Gentzsch et al, 2018; Chen et al, 2019). In contrast to independent factors, a combination of NR/IL-1*β* enabled detection of VX-809/VX-770 modulator responses in CF F508del/F508del NAOs (Figs 2H and S3B). The combined effect is likely required because of the activation of distinct signaling transduction pathways, that is, mitogen-activated protein kinases and NF-κB, respectively, based on studies in ALI cultures (Brouillard et al, 2001; Kettle et al, 2010). In contrast to CF NAOs, NR/IL-1*β* did not reduce FIS measured in HC NAOs (Fig S3C and D). Corresponding with reduced CFTR-independent fluid secretion, NR/IL-1*β* attenuated lumen formation in CF NAOs (Fig S4A and B). Moreover, in line with enhanced specificity of CFTR function, NR/IL-1*β* increased and reducing the expression of *CFTR* and the CaCC *ANO1*, respectively (Fig S4C). The Cl^−^ channel *SLC26A9* was also increased upon stimulation with NR/IL-1*β*, which may support CFTR protein stability or function, as proposed by others (Balázs & Mall, 2018). NR/IL-1*β* did not affect the expression of ciliated and goblet cells markers (Fig S4D and E), suggesting that increased *CFTR* and *SLC26A9* expression is not caused by alterations in mucociliary differentiation. To further demonstrate CFTR dependence of FIS in the NR/IL-1*β* culture condition and to exclude potential inadequate inhibition of CFTR with chemical inhibitors earlier used, we generated *CFTR* gene KO HNEC using CRISPR gene editing (Fig S5A). WT and *CFTR* KO cells were differentiated in ALI cultures, converted into NAOs, and cultured with NR/IL-1*β*, which attenuated lumen formation (Fig S5B). *CFTR* KO NAOs displayed attenuated FIS compared with WT controls (Figs 2I and S5C). In contrast, comparable swelling was observed in response to E_act_ (Figs 2J and S5D). This corresponded with persistent E_act_-induced swelling in CF NAOs cultured with NR/IL-1*β* (Fig S5E and F). Altogether, studies in *CFTR* KO NAOs further confirmed CFTR dependence of FIS in the NR/IL-1*β* culture condition.

**Figure 3.**
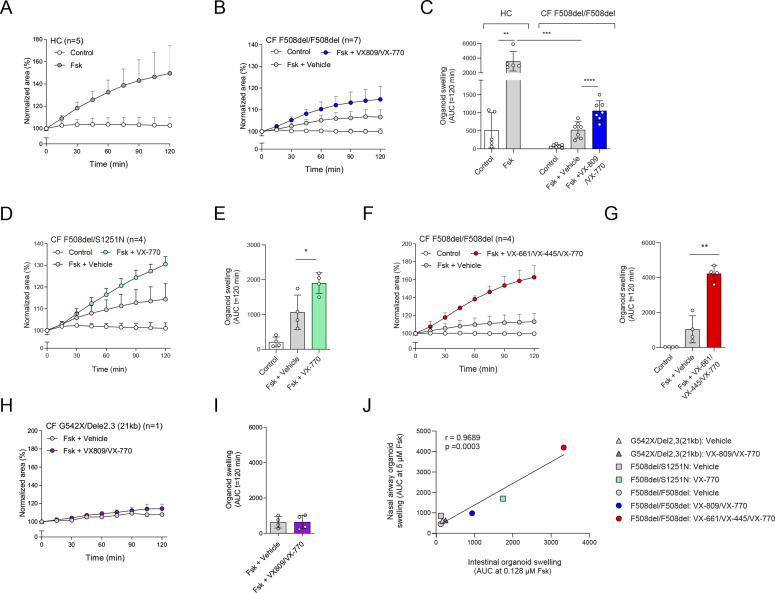
Validation of CFTR modulator responses in NR/IL-1*β*–cultured nasal-airway organoids (NAOs). **(A, B)** Forskolin-induced swelling (FIS) measured in HC NAOs (n = 5 independent donors) and (B) cystic fibrosis (CF) F508del/F508del NAOs (n = 7 independent donors) cultured with NR/IL-1*β*. FIS responses in CF NAOs were determined in response to VX-809/VX-770. **(C)** Comparison of FIS measured in HC (Fig 3F) and CF F508del/F508del NAOs (Fig 3E). **(D, E)** FIS in NR+IL-1*β* cultured NAOs of CF F508del/S1251N subjects (n = 4 independent subjects), stimulated with Fsk and VX-770 or vehicle. **(F, G)** CF F508del homozygous NAOs cultured with NR/IL-1*β* (n = 4 independent donors) were pre-treated with vehicle or VX-661/VX-445. Swelling was determined afterward following acute stimulation with Fsk together with VX-770 or vehicle. **(H, I)** FIS in NR+IL-1*β*-cultured NAOs of an individual with CF and a severe R553X/R553X genotype. Organoids were pre-treated with VX-809 or vehicle. Swelling was determined afterward following acute stimulation with Fsk with or without VX-770. **(J)** Pearson correlation between FIS measured in NAOs and CFTR genotype–matched intestinal organoids. **(A, B, C, D, E, F, G, H, I, J)** Data information: swelling results are depicted as (A, B, D, F, H); the percentage change in surface area relative to t = 0 (normalized area) measured at 15-min time intervals for 2 h (means ± SD) and (C, E, G, I, J), area-under-the-curve (AUC) plots (t = 120 min, means ± SD), datapoints represent individual donors (C, E, G) or technical replicates (I). Correlation between nasal and intestinal organoid FIS was examined with AUC values at 5 and 0.128 μM Fsk, respectively. **(C, E, G, I, J)** Analysis of differences was determined with a paired *t* test (C; within groups, E, G, I), unpaired *t* test (C; HC compared with CF), and Pearson correlation (J). *P < 0.05, **P < 0.01, ***P < 0.001, ****P < 0.0001.

**Figure S6.**
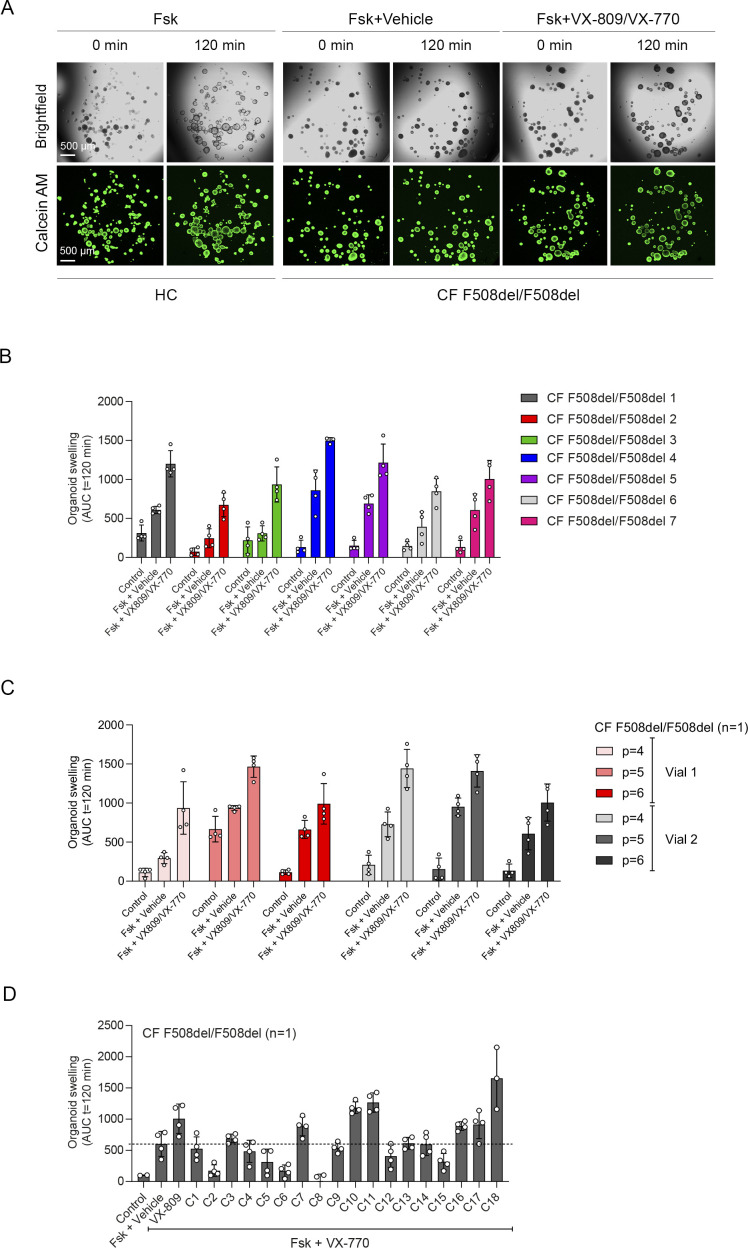
Comparison HC versus cystic fibrosis (CF) nasal-airway organoids (NAOs) cultured with NR/IL-1*β* and validation of VX-809/VX-770 responses. **(A)** Brightfield images (top) and images of calcein green AM–stained (bottom) NAOs cultured with NR/IL-1*β*, showing forskolin-induced swelling (FIS) in HC and CF F508del/F508del cultures. FIS of CF NAOs was determined in combination with vehicle or VX-809/VX-770. Images were taken at t = 0 and 120 min. **(B)** VX-809/VX-770 modulator response measurement in NAOs of individual donor (n = 7) with a CF F508del/F508del genotype. **(C)** Repeated measurements of VX-809/VX-770 modulator responses in CF F508del/F508del NAOs (n = 1 independent donor) derived from air–liquid interface cultures from serial passaged human nasal epithelial cell at *P* = 4, *P* = 5, and *P* = 6, using cryopreserved cells from two different vials. **(D)** CF F508del/F508del NAOs (n = 1 independent donor) were pre-treated with VX-809 or the CFTR correctors C1-18 for 48 h. Afterward, FIS was measured after stimulation with forskolin (Fsk) and VX-770. Vehicle was used as the control. FIS assay results are depicted as area-under-the-curve (AUC) plots (t = 120 min), means ± SD. Individual datapoints represent technical replicates.

**Figure S7.**
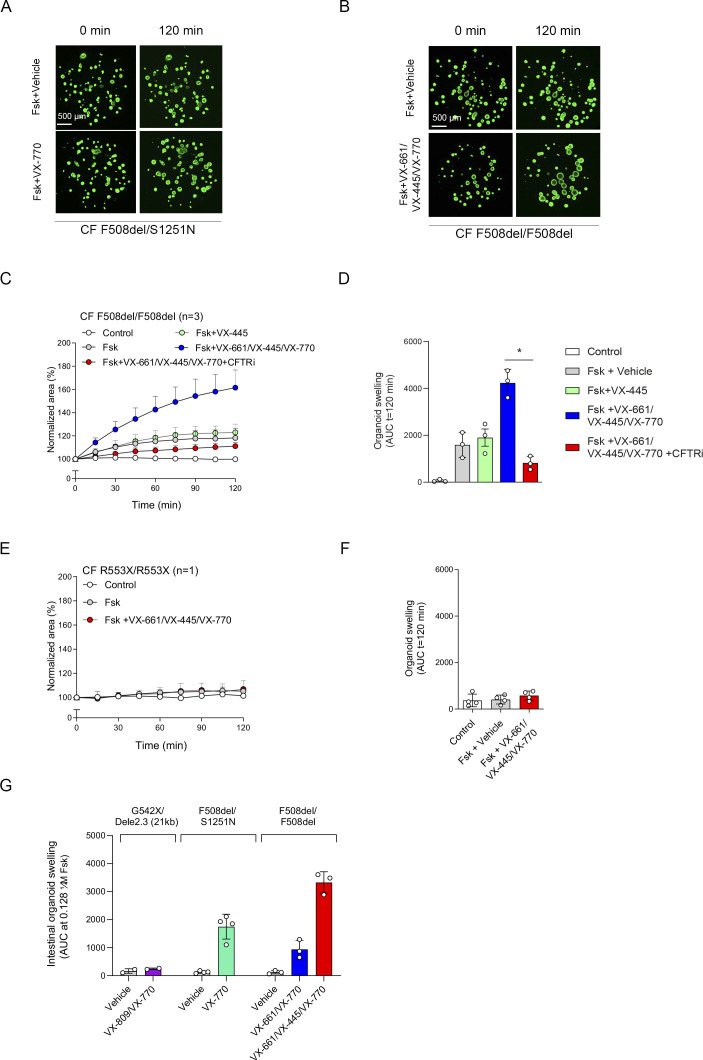
Validation of VX-770, VX-661/VX-445/VX-770, and forskolin-induced swelling (FIS) in intestinal organoids. **(A)** Images showing FIS of NR+IL-1*β* cultured nasal-airway organoids (NAOs) from individuals with cystic fibrosis (CF) and F508del/S1251N genotype. NAOs were stimulated with Fsk and vehicle (top panels) or VX-770 (bottom panels). **(B)** Images of FIS conducted in calcein green AM–stained CF F508del/F508del NAOs cultured with NR/IL-1*β*, pre-treated with vehicle (top panels) or triple CFTR modulator combination VX-661/VX-445/VX-770 (bottom panels). Images were taken at t = 0 and 120 min. **(C, D)** CF F508del homozygous NAOs (n = 3 independent donors) were pre-treated with vehicle, VX-445, or a combination of VX-661 and VX-445 for 48 h. Next, cultures were pre-treated with CFTR inhibitors CFTRinh-172 and GlyH101 or vehicle for 4 h, followed by stimulation with Fsk together with VX-770 or vehicle as indicated. **(E, F)** R553X/R553X NAOs (n = 1 independent donor) were pre-treated with vehicle or a combination of VX-661 and VX-445 for 48 h, followed by assessment of FIS after stimulation with Fsk together with VX-770 or vehicle as indicated. **(G)** FIS measured with indicated CFTR modulators in intestinal organoids from individuals with CF and G542X/Dele2,3(21 kb) (n = 2 independent donors), F508del/S1251N (n = 4 independent donors), F508del/F508del (n = 3 independent donors) genotypes, used for Pearson correlation in Fig 3J. **(C, D, E, F, G)** Results are depicted as percentage increase in normalized area in time (means ± SD) (C, E) and AUC plots (t = 120 min, means ± SD) (D, F, G). **(D, F, G)** Datapoints represent individual donors (D, G) or technical replicates (F). **(D)** Analysis of differences was conducted with a paired *t* test (D) *P < 0.05.

Next, we further validated CFTR function and modulator responses in the NR/IL-1*β* organoid culture condition. Similar to cultures without NR/IL-1*β* (Fig 2C and D), HC NAOs displayed a significant higher swelling response when compared with cultures from CF F508del/F508del subjects (Figs 3A–C and S6A). Moreover, the CFTR modulator combination VX-809/VX-770 consistently enhanced FIS measured in CF F508del/F508del NAOs from seven independent subjects (Figs 3B and C and S6B). In addition, consistent responses to VX-809/VX-770 were observed in CF F508del/F508del NAOs derived from the same donor, differentiated in ALI cultures at passage 4–6, and derived from two separate cryopreserved vials from the same work cell bank (Fig S6C). In addition to VX-809, FIS in CF F508del/F508del NAOs were selectively modulated upon treatment with other CFTR correctors (Fig S6D). Moreover, genotype-specific VX-770 potentiator responses were observed in NAOs from subjects with CF and a S1251N gating mutation (Figs 3D and F and S7A). The CFTR triple modulator therapy VX-445/VX-661/VX-770 (Keating et al, 2018), induced a high increase in FIS in CF F508del/F508del NAOs cultured with NR/IL-1*β* (Figs 3F and G and S7B). VX-445 by itself did not increase FIS in CF F508del/F508del NAO, whereas chemical CFTR inhibition completely diminished increases in swelling by VX-445/VX-661/VX-770 (Fig S7C and D). VX-445/VX-661/VX-770 did not improve FIS in NAOs from an individual with CF and a severe R553X/R553X genotype (Fig S7E and F). Altogether, these findings demonstrate robust increases in FIS by VX-445/VX-661/VX-770 in a CFTR specificity–specific manner.

As a final validation study, we determined the correlation between FIS in NAOs from individuals with CF and FIS in CFTR genotype–matched intestinal organoids (Fig S7G). We compared the effect of VX-770 in F508del/S1251N organoids (Fig 3E), VX-809/VX-770 (Fig 3C), and VX-445/VX-661/VX-770 (Fig 3G) in F508del/F508del organoids and the effect of VX-809/VX-770 in organoids of individuals with a severe G542X/Dele2,3(21 kb) genotype (Fig 3H and I). We observed a strong correlation (r = 0.9689, *P* = 0003) between FIS in CF NAOs and intestinal organoids (Fig 3J). This suggests that CFTR function and modulator responses in NAOs are comparable to the CFTR-dependent intestinal organoid model.

**Figure S8.**
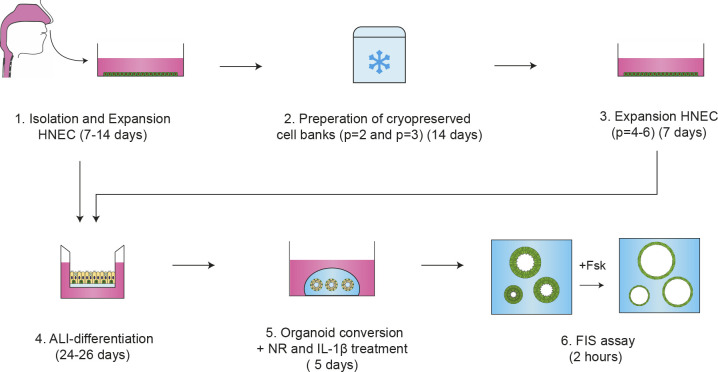
Graphic illustration of the whole timeline from nasal-cell isolation till forskolin-induced swelling assay in nasal-airway organoids.

In summary, we described a new method of culturing nasal-brushing–derived airway organoids, which can be used to determine CFTR modulator responses in individuals with CF. Starting with a nasal brush till assessment of CFTR modulator response in NAOs with FIS (Fig S8), the procedure takes ∼57–66 d, varying between donors. However, for personalized medicine purposes, the procedure can be reduced to 36–45 d, by excluding de generation of cryopreserved cell banks and instead using freshly isolated cells. Previously, we have shown that long-term expanded airway organoids can be cultured as 2D differentiated ALI cultures (Sachs et al, 2019). In this report, we demonstrate further flexibility between 2D and 3D airway culture models, by showing the possibility to convert 2D differentiated ALI cultures into 3D organoids. We furthermore described airway organoid culture conditions that improved quantification of CFTR modulator responses in FIS assays. The NR/IL-1*β* culture condition may reflect the chronically inflamed airway epithelium of individuals with CF and therefore may act as physiological condition for testing CFTR modulator responses in NAOs. Further research is required to determine whether NR/IL-1*β* also improve CFTR modulator responses in other airway model systems, such as distal airway organoids, or other CFTR-expressing epithelial cells.

Limitation of our model is that we cannot discriminate HC from CF NAOs based on steady-state lumen size, because of CFTR-independent fluid secretion, which is in contrast to the CFTR-dependent intestinal organoid model (Dekkers et al, 2013, 2016). Furthermore, we are unable to use CFTR modulator response measurements in NAOs to estimate the level of CFTR repair that is achieved as compared with HC activity, as exemplified by comparable FIS in HC NAOs and F508del/F508del CF NAOs treated with VX-445/VX-661/VX-770 (Fig 3C and G). Furthermore, comparison studies remain required to determine whether CFTR modulator responses based on short-circuit current measurements conducted in ALI cultures, correlate with organoid swelling, as recently shown by others (Anderson et al, 2021; Sette et al, 2021).

Indeed, we observed a correlation between CFTR modulator responses in nasal and intestinal organoids. FIS in intestinal organoids is fully CFTR-dependent, and previously, it has been shown that CFTR modulator responses in intestinal organoids correlate with drug efficacy in individuals with CF (Berkers et al, 2019; Ramalho et al, 2020; Muilwijk et al, 2022). Therefore, a correlation between nasal and intestinal organoids provides early evidence that FIS in NAOs can also be used to predict CFTR modulator efficacy in a genotype-dependent manner. However, the potential clinical impact of our method requires follow-up studies, such as further exploration whether CFTR modulator responses in ALI-derived NAOs correlate with the clinical outcome in a large cohort of individuals with CF and how this relates to other in vitro measurements, that is, patient-matched intestinal organoids and 2D ALI airway cultures. CFTR function measurements in ALI culture–derived NAOs may also be used to examine novel target-specific therapies for subjects with CF with unmet, such as assessment of CRISPR gene editing, read-through agents, or compounds targeting nonsense-mediated decay. Moreover, complementary to the widely used 2D ALI cultures, airway organoids derived from this model may be further used to study other respiratory tract disorders.

## Materials and Methods

**Table:** Reagents and tools table.

Reagent or resource	Source	Identifier
Antibodies		
Mouse IgG1 anti-*β*-tubulin IV	Emergo Biogenex	#MU178-UC
Rabbit anti-*β*-tubulin IV	Abcam	#ab179509
Rabbit anti-MUC5AC	Abcam	#ab198294
Rabbit anti-P63	Abcam	#ab124762
Mouse IgG1 anti cytokeratin 5	Abcam	#ab17130
Mouse IgG1 anti-CC10	Acris, Origene	#AM26360PU-N
Goat anti-Mouse IgG1, Alexa Fluor 488	Invitrogen	#A-21121
Goat anti-Mouse IgG1, Alexa Fluor 647	Invitrogen	#A-21240
Goat anti-Rabbit IgG, Alexa Fluor 488	Invitrogen	#A-11034
Goat anti-Rabbit IgG, Alexa Fluor 546	Invitrogen	#A-11035
Biological samples		
Nasal brushings	UMC Utrecht	Protocol ID: 16/586, NL54885.041.16
Nasal turbinate tissue	Academic Medical Center Amsterdam	N/A
Intestinal organoids	HUB	https://huborganoids.nl/
Chemicals, peptides, and recombinant proteins		
TrypLE express enzyme	Thermo Fisher Scientific	# 12605010
Sputolysin	Calbiochem	#560000-10
Collagen IV	Sigma-Aldrich	#C7521
CryoStor CS10	STEMCELL Technologies	#07930
PureCol	Advanced BioMatrix	#5005
Bronchial epithelial cell medium-basal (BEpiCM-b)	ScienCell	#3211
Advanced DMEM F12	Thermo Fisher Scientific	#12634-028
B-27 Supplement, serum free	Thermo Fisher Scientific	#12587010
GlutaMAX Supplement	Thermo Fisher Scientific	#35050-061
HEPES	Thermo Fisher Scientific	#15630080
(±)-Epinephrine hydrochloride	Sigma-Aldrich	#E4642
Hydrocortisone	Sigma-Aldrich	#H0888
3,3′,5-Triiodo-L-thyronine sodium salt	Sigma-Aldrich	#T6397
N-Acetyl-L-cysteine	Sigma-Aldrich	#A9165
Nicotinamide	Sigma-Aldrich	#N0636
SB02190	Sigma-Aldrich	#S7067
DMH-1	Selleck Chemicals	#S7146
A83-01	Tocris	#2939/10
Y-27632	Selleck Chemicals	#S1049
DAPT	Thermo Fisher Scientific	#15467109
TTNPB	Cayman	#16144-1
Recombinant human FGF-7	PeproTech	#100-19
Recombinant human FGF-10	PeproTech	#100-26
Recombinant human EGF	PeproTech	#AF-100-15
Recombinant human HGF	PeproTech	#100-39H
Recombinant Neuregulin-1*β*	PeproTech	#100-03
Recombinant Interleukin 1*β*	PeproTech	#200-01B
Penicillin–Streptomycin	Thermo Fisher Scientific	#15070-063
Primocin	InvivoGen	#ant-pm-2
Amphotericin B	Thermo Fisher Scientific	#15290018
Gentamicin	Sigma-Aldrich	#G1397
Vancomycin	Sigma-Aldrich	#SBR00001
Collagenase type II	Thermo Fisher Scientific	#17101-015
CFTR multi-guide sgRNA	Synthego	
2NLS-Cas9 nuclease	Synthego	
OptiMEM	Thermo Fisher Scientific	#31985062
CFTRinh-172	Sigma-Aldrich	#C2992
GlyH101	Sigma-Aldrich	#219671
VX-809	Selleck Chemicals	#S1565
VX-661	Selleck Chemicals	#S7059
VX-770	Selleck Chemicals	#S1144
Correctors C1-18	Cystic Fibrosis Foundation Therapeutics	https://www.cff.org/Research/Researcher-Resources/Tools-and-Resources/CFTR-Chemical-Compound-Program/
Forskolin	Sigma-Aldrich	#F3917
3-Isobutyl-1-methylxanthine	Sigma-Aldrich	#I5879
Amiloride	Sigma-Aldrich	#1019701
Calcein green acetoxymethyl (AM)	Invitrogen	#C34852
Alexa Fluor 555 Phalloidin	Invitrogen	#A34055
Phalloidin-iFluor 405	Abcam	#ab176752
ProLong Gold antifade reagent	Thermo Fisher Scientific	#P36934
ProLong Gold antifade reagent with DAPI	Thermo Fisher Scientific	#P36935
iQ SYBR Green Supermix	Bio-Rad	#1708880
Critical Commercial Assays		
RNeasy Mini Kit	QIAGEN	#74104
iScript cDNA synthesis kit	Bio-Rad	#1708891
Quick-DNA Microprep Kit	Zymo Research	#D3020
GoTaq G2 Flexi DNA polymerase	Promega	#M7805
Experimental Models: Cell Lines		
Human nasal airway epithelial cells (HNEC)	This paper	N/A
Hek293T – R-spondin-1 mFc cell line	Trevigen Cat. no. 3710-001-	N/A
Oligonucleotides		
Primers used for this manuscript		Table S5
Equipment		
Zeiss LSM800 confocal microscopy	Zeiss	N/A
Leica SP8X confocal microscope	Leica	N/A
Leica THUNDER imager	Leica	N/A
Leica TCS SP8 STED 3X microscope	Leica	N/A
NEPA21	NEPA	N/A
Ussing chamber system	Physiologic Instruments	N/A
Voltage clamp	World Precision Instruments	#DVC-1000
PowerLab	AD Instruments	#8/30
NanoDrop spectrophotometer	Thermo Fisher Scientific	N/A
CFX96 real-time detection machine	Bio-Rad	N/A
Software and Algorithms		
Zen Blue Software	Zeiss	https://www.zeiss.com/microscopy/int/products/microscope-software/zen.html
Prism 8	GraphPad Software Inc.	https://www.graphpad.com/scientific-software/prism/
Microsoft Excel	Microsoft Corporation	https://office.microsoft.com/excel
Adobe Illustrator	Adobe	https://www.adobe.com/nl/products/illustrator.html
LabChart 6	AD Instruments	https://labchart.software.informer.com/6.0/
CFX Manager 3.1	Bio-Rad	https://www.bio-rad.com/en-us/sku/1845000-cfx-manager-software?ID=1845000
LAS X software	Leica	https://www.leica-microsystems.com/products/microscope-software/p/leica-las-x-ls/
ImageJ/FIJI		https://imagej.net/Fiji/Downloads
ICE analysis tool	Synthego	https://ice.synthego.com/#/
Others		
Cytological brush	CooperSurgical	#C0004
12-mm Transwell with 0.4-*μ*m Pore Polyester Membrane Insert	Corning	#3460
6.5-mm Transwell with 0.4-*μ*m Pore Polyester Membrane Insert	Corning	#3470
Matrigel	Corning	#354230
BC isolation and expansion medium		Table S2
Air–liquid interface differentiation medium		Table S3
Airway organoid culture medium		Table S4

### Patient materials and sample collection

Nasal brushings were collected from healthy volunteers without respiratory tract symptoms (n = 19 independent donors) and subjects with CF (n = 24 independent donors) by a trained research nurse or physician, essentially as previously described (Harris et al, 2004). The use of donor cells in different experiments depicted in the figures has been described in Table S1. Sampling of adults was conducted using a cytological brush and without anesthetics. Nasal brushings of infants (<6 yr old) were collected with a modified interdental brush (Stokes et al, 2014). Samples were taken from the inferior turbinates of both left and right nostrils and stored in collection medium, consisting of advanced (ad)DMEM/F12, with GlutaMAX (1% vol/vol), Hepes (10 mM), penicillin–streptomycin (1% vol/vol), and Primocin (100 μg/ml). Nasal brushings were collected and stored with informed consent of all participants and was approved by a specific ethical board for the use of biobanked materials TcBIO (Toetsingscommissie Biobanks), an institutional Medical Research Ethics Committee of the University Medical Center Utrecht (protocol ID: 16/586). Nasal samples from infants with CF were collected as part of the Precision study (protocol ID: NL54885.041.16), which was approved by the Medical Research Ethics Committee of the University Medical Center Utrecht. Intestinal organoids (n = 9 independent donors) were collected, generated, and stored with informed consent of all participants and was approved by the TcBIO (UMCU; TcBio#14-008) according to the guidelines of the European Network of Research Ethics Committees (EUREC). Biobanked intestinal organoids are stored and cataloged (https://huborganoids.nl/) at the foundation Hubrecht Organoid Technology (http://hub4organoids.eu). Resected inferior nasal turbinate tissue was obtained from a subject who underwent corrective surgery for turbinate hypertrophy at the Academic Medical Center in Amsterdam, the Netherlands. The tissue was accessible for research within the framework of patient care, in accordance with the “Human Tissue and Medical Research: Code of conduct for responsible use” (2011), describing the no-objection system for coded anonymous further use of such tissue without necessary written or verbal consent.

Table S1 Use of human nasal epithelial cell donors in the depicted figures.

### Isolation and expansion of human nasal airway epithelial cells as 2D-cultures

Nasal cells were dissociated from the brush in the collection medium by scraping through a sterile P1000 pipette tip with the top cut off. After centrifugation (400*g* for 5 min), the pellet was treated with the TrypLE express enzyme, supplemented with 1× Sputolysin. The cells were incubated for 10 min at 37°C and strained using a 100-μm strainer. After centrifugation (400*g* for 5 min), the remaining pellet was used for isolation of HNECs. HNECs were isolated and expanded on six-well culture plates coated with collagen IV (50 μg/ml) and using BC isolation and expansion medium (Table S2), respectively. BC isolation medium contained additional antibiotics to suppress microbial outgrowth and was used during the first week of epithelial cell isolation, before switching to BC expansion medium. BC expansion medium included the γ-secretase inhibitor DAPT, which reduces outgrowth of squamous cells at late passages. Growth factors (FGF7, FGF10, EGF, and HGF) were added freshly to the culture medium. Cultures were refreshed three times a week (Monday, Wednesday, and Friday) and passaged after reaching 80–90% confluency, typically within 7–14 d. This varied between donors and mainly depending on the number of cells harvested during brushings. During passaging, cells were dissociated with the TrypLE express enzyme. Isolated cells (passage 1) were expanded for an additional 7 d, before freezing with CryoStor CS10, supplemented with Y-27632 (5 μM) to create a master cell bank (passage 2). For further use, HNEC were expanded (∼7) d and cryo-stored as a work cell bank (passage 3).

Table S2 Composition of BC isolation and expansion medium.

### Differentiation of 2D ALI-HNEC cultures

HNEC (passage 4–6) were cultured on transwell inserts (0.4-μm pore size polyester membrane), which were coated with PureCol (30 μg/ml). Cells were seeded in a density of 0.2 or 0.5 × 10^6^ cells on 24- or 12-well inserts, respectively, and cultured in submerged conditions in BC expansion medium until reaching confluency after 3–5 d. Afterward, culture medium was changed with ALI-differentiation medium (Table S3) supplemented with A83-01 (500 nM), and cells were in addition cultured in submerged condition for 1–2 d. Subsequently, culture medium at the apical side was removed and cells were further differentiated as ALI cultures. After 3–4 d, cells were refreshed with ALI-diff medium without additional A83-01 and differentiated for at least 14 additional days at ALI-conditions. Medium was refreshed twice a week (Monday and Thursday or Tuesday and Friday), and the apical side of the cultures was washed with PBS once a week.

Table S3 Composition of 2D air–liquid interface differentiation medium.

### Conversion of 2D differentiated ALI cultures into airway organoids

Differentiated ALI cultures were washed at the apical surface with PBS and subsequently treated at the basolateral side with collagenase type II (1 mg/ml) diluted in adDMEM/F12. Cultures were incubated at 37°C and 5% CO_2_ for 45–60 min until the epithelium detaches from the transwell insert. Next, the dissociated epithelial layer was transferred to a 15-ml tube in 1 ml adDMEM/F12 + 10% (vol/vol) FBS, mechanically disrupted into smaller fragment by pipetting, and strained with a 100-μm filter. After centrifugation (at 400*g*, 5 min), the epithelial pellet was resuspended in ice-cold 75% growth factor reduced Matrigel (vol/vol in airway organoid [AO] medium Table S4). Next, epithelial fragments were embedded in 30 μl Matrigel droplets on pre-warmed 24-well suspension plates. Droplets were solidified at 37°C and 5% CO_2_ for 20–30 min, before adding 0.5 ml AO medium (Table S4). In optimized conditions for measuring CFTR modulator responses, AO culture medium was further supplemented with neuregulin-1*β* (NR, 0.5 nM) and interleukin-1*β* (IL-1*β*; 10 ng/ml). Besides, NR/IL-1*β* we furthermore examined the effects of culturing with other interleukins, that is, IL-13, IL-4, IL-10, and the growth factors: fibroblast growth factor 2, 7, 10, hepatocyte growth factor, and insulin-like growth factor 1, which did not improve the detection of CFTR modulator responses. AO medium was refreshed twice a week (Monday and Thursday or Tuesday and Friday).

Table S4 Composition of airway organoid medium.

### FIS assay

ALI-derived NAOs were used in FIS assays, essentially as previously described with minor adaptation (Boj et al, 2017; Sachs et al, 2019). In short, organoids were transferred 3–5 d after conversion in 96-well plates in 4 μl droplets of 75% Matrigel (vol/vol in AO medium), containing ∼25–50 structures. After solidification of droplets, 100 μl AO medium was added to each well. In optimized conditions for measuring CFTR modulator responses, AO culture medium was supplemented with neuregulin-1*β* (0.5 nM) and interleukin-1*β* (10 ng/ml). Organoid swelling was conducted with four technical replicates. In indicated experiments, organoids were pre-treated with CFTRinh-172 and GlyH101 (CFTRi, both 50 μM) or vehicle as negative control for 4 h. CFTR correctors: VX-809, VX-661 (both 10 μM), VX-445 (5 μM), C1-18 (all 10 μM), or vehicle were pre-treated for 48 h. Before assessment of FIS, NAOs were stained with calcein green AM (3 μM) for 30 min. Afterward, organoids were stimulated with forskolin with indicated concentration. In cultures from subjects with CF, the CFTR potentiator VX-770 (10 μM) or vehicle was added together with forskolin. Swelling of NAOs was quantitated by measuring the increase of the total area of calcein green AM–stained organoids in a well during 15-min time intervals for a period of 2 h. Images were acquired with a Zeiss LSM800 confocal microscopy, using a 2.5 or 5× objective, and experiments were conducted at 37°C and 95% O_2_/5% CO_2_ to maintain a pH of 7.4. Data were analyzed using Zen Blue Software and Prism 8.

### Ussing chamber experiments

For open circuit Ussing chamber measurements, transwell inserts (⌀12 mm) were mounted in the chamber device and continuously perfused at the apical and basal side with a Ringer solution of the following composition (mmol/l) 145 NaCl, 1.6 K_2_HPO_4_, 1 MgCl_2,_ 0.4 KH_2_PO_4_, 1.3 Ca^2+^ gluconate, and 5 glucose and pH adjusted to 7.4. After a 20-min stabilization period, amiloride (20 μM) was added to the apical side to block epithelial Na^+^ channel–mediated currents, followed by forskolin/IBMX (2 μM/100 μM), VX-770 (3 μM), and CFTRInh-172 (30 μM) were added sequentially. Transepithelial voltage (V_te_) values were recorded at all times with PowerLab software (AD Instruments Inc.). Values for V_te_ were referred to the basal side of the epithelium, and transepithelial resistance (R_te_) was determined by applying short intermittent pulses (0.5 μA/s), measuring pulsated deviations in V_te_ and accounting for the area of the inserts. An empty insert was previously recorded to correct the measured values. Short-circuit currents (I_eq-sc_) were calculated according to Ohm’s law from V_te_ and R_te_ (I_eq-sc_ = V_te_/R_te_).

### CRISPR gene editing

CFTR gene KO HNEC (n = 3 independent donors) were generated by electroporation of recombinant Cas9/single-guide RNA RNP complexes. RNP complexes were prepared by mixing multi-guide single-guide RNA (30 μM, 8.3 μl), recombinant 2NLS-Cas9 nuclease (20 μM, 2.5 μl), and 14.2 μl OptiMEM supplemented with 10 μM Y-27632, followed by incubation at room temperature for 10 min. After expansion, HNEC (passage 3) were dissociated into single cells using the TrypLE express enzyme. Next, 1 × 10^6^ cells were diluted in 75 μl optiMEM with 10 μM Y-27632 and added to the RNP complexes. Electroporation was conducted with the NEPA21 according to previously published settings (Fujii et al, 2015). After electroporation, HNEC were seeded in 12-well plates in BC expansion medium. After expansion, HNEC were used for further experiments. For assessment of KO efficiency, DNA was isolated according to the manual of the Quick-DNA Microprep Kit. Regions of interest were amplified in a PCR reaction with GoTaq G2 Flexi DNA polymerase with primers, and PCR-amplified samples were run on 1.2% TBE-agarose gel for size separation. DNA fragments were excised from the gel, purified according to the gel extraction kit, and sent for Sanger sequencing with sequencing primers. Analysis of the KO efficiency and the specific deletions were done with the ICE analysis tool (https://ice.synthego.com/#/). Of independent donors, the calculated CFTR KO efficiency was 69%, 76%, and 84%, respectively.

### RNA extraction, cDNA synthesis, and quantitative real-time PCR

Total RNA was extracted from ALI cultures using the RNeasy Mini Kit according to the manufacturer’s protocol. RNA yield was determined by a NanoDrop spectrophotometer, and subsequently, cDNA was synthesized by use of the iScript cDNA synthesis kit according to the manufacturer’s protocol. Quantitative real-time PCR (qPCR) was performed with specific primers (Table S5) using the iQ SYBR Green Supermix and a CFX96 real-time detection machine. CFX Manager 3.1 software was used to calculate relative gene expression normalized to the housekeeping genes *ATP5B* and *RPL13A*, according to the standard curve method. Housekeeping genes were selected based on stable expression in airway epithelial cells at different experimental conditions, based on the geNorm method (Vandesompele et al, 2002).

Table S5 qPCR primer sequences.

### Immunofluorescence staining and microscopy

2D expanded HNEC and ALI-HNEC cultures were fixed in 4% paraformaldehyde for 15 min, permeabilized in 0.25% (vol/vol) Triton-X in PBS for 30 min and treated with blocking buffer, consisting of 1% (wt/vol) BSA, and 0.25% (vol/vol) Triton-X in PBS for 60 min. Next, primary antibodies (1:500) in blocking buffer were added at the apical side and incubated for 1–2 h or overnight. Afterward, cells were washed three times with PBS and incubated with secondary antibodies and phalloidin (1:500) in blocking buffer for 30 min in dark, followed by three washings in PBS. Transwell membranes were subsequently cut from the inserts and placed on slides. All samples were mounted with the ProLong Gold antifade reagent with or without DAPI. Resected nasal tissue and ALI-HNEC cultures were fixed with 4% paraformaldehyde and embedded in paraffin after dehydration. After deparaffinization followed by antigen retrieval using citrate buffer (pH = 6) for 20 min, the 5-μm sections were permeabilized in 0.25% (vol/vol) Triton-X in PBS for 15 min, then treated with blocking buffer, consisting of 5% (wt/vol) BSA and 0.025% (vol/vol) Triton-X in PBS, for 30 min. Primary antibodies in blocking buffer were incubated for 2 h, followed by incubation of secondary antibodies for 1 h. Samples were mounted with ProLong Gold reagent with DAPI. Organoids plated in 4 μl droplets of 75% Matrigel (vol/vol) in a 96-well plate were fixed with 4% paraformaldehyde for 10 min and stained as previously described (Dekkers et al, 2019; Sachs et al, 2019), using indicated primary antibodies. Images were acquired with a Leica SP8X confocal microscope, Leica THUNDER imager, and Leica TCS SP8 STED 3X microscope. Images were processed using LAS X software and ImageJ/FIJI.

### Intestinal organoid culturing and use in FIS assays

Intestinal organoids of individuals with CF were isolated from rectal biopsies, expanded, and used in FIS assays as previously described (Dekkers et al, 2013; Vonk et al, 2020). For FIS assays, intestinal organoids were plated in 96 wells and preincubated with vehicle or indicated CFTR correctors, VX-809, VX-661 (both 3 μM), and VX-445 (5 μM) for 24 h. FIS was measured upon addition of forskolin (0.128 μM) and the CFTR potentiator VX-770 (3 μM) for 60 min. A forskolin concentration of 0.128 μM was used, based on previous correlation studies with clinical outcome measurements (Berkers et al, 2019).

### Quantification and statistical analysis

Swelling assays were conducted with four technical replicates for each experimental condition, and results are shown as mean ± SD of independent subjects or independent technical replicates as indicated in the figure legends. Increases in the total surface area of all organoids in a single well is calculated as normalized swelling, relative to t = 0, which is set as baseline of 100%. For swelling assays, statistical analysis was assessed with area under the curve (AUC) values (t = 120 min). Analysis of differences was determined with a one/two-way repeated measurements ANOVA and Bonferroni post hoc test or (un)paired *t* test as indicated in the figure legends. Normal distribution was tested using the Shapiro–Wilk test. Differences were considered significant at *P* < 0.05. Statistical analysis was conducted using Prism 8 (GraphPad Software Inc.).

## Data Availability

All data are provided with the manuscript.

## Supplementary Material

Reviewer comments
